# Three New Pyridine Alkaloids from *Vinca major* Cultivated in Pakistan

**DOI:** 10.1007/s13659-017-0137-7

**Published:** 2017-06-15

**Authors:** Xin Wei, Afsar Khan, Da Song, Zhi Dai, Ya-Ping Liu, Hao-Fei Yu, Bei Wang, Pei-Feng Zhu, Cai-Feng Ding, Xu-Dong Zhao, Yi-Fen Wang, Xiao-Dong Luo

**Affiliations:** 1 State Key Laboratory of Phytochemistry and Plant Resources of West China, Kunming Institute of Botany, Chinese Academy of Sciences, Kunming, 650201 People’s Republic of China; 2 Yunnan College of Business Management, Kunming, 650106 People’s Republic of China; 3 Department of Chemistry, COMSATS Institute of Information Technology, Abbottabad, 22060 Pakistan; 4 Key Laboratory of Animal Models and Human Disease Mechanisms, Chinese Academy of Sciences & Yunnan Province, Kunming Institute of Zoology, Kunming, 650223 Yunnan People’s Republic of China; 5 University of Chinese Academy of Sciences, Beijing, 100049 People’s Republic of China; 6 Yunnan Key Laboratory of Natural Medicinal Chemistry, Kunming, 650201 People’s Republic of China

**Keywords:** *Vinca major*, Apocynaceae, Pyridine alkaloids, Cytotoxicity

## Abstract

Three new pyridine type alkaloids, (−)-vinmajpyridines A–C (**1**–**3**), along with two known alkaloids, have been isolated from the aerial parts of *Vinca major* cultivated in Pakistan. Their structures have been elucidated by means of NMR and HRESIMS spectroscopic data. The new alkaloids were evaluated for their cytotoxicity against glioma initiating cell lines (GITC-3^#^ and GITC-18^#^), glioblastoma cell lines (U-87MG and T98G), and lung cancer cell line A-549, but none of them was active at 20 μg/mL concentration.

**Electronic supplementary material:**

The online version of this article (doi:10.1007/s13659-017-0137-7) contains supplementary material, which is available to authorized users.

## Introduction

**Fig. 1:**
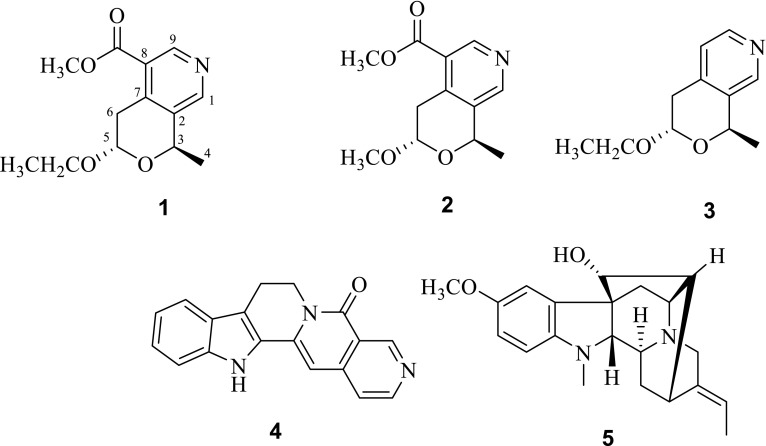
Structures of compounds **1**–**5**

The genus *Vinca* (Apocynaceae), distributed through Europe, Northwest Africa, and South-west Asia, represents a group of species which are rich in indole alkaloids of diverse structural patterns, many of which are of considerable therapeutic value [1–4]. *Vinca major* has been used for centuries as a folk remedy in the treatment of menorrhagia and diabetes, and as an abortifacient and vulnerary [5]. Delphinidin glycosides have been isolated from the flowers of *V. major* [6] while chlorogenic acid, robinin, and flavonol triglycoside are extracted from its leaves [7]. In addition, a number of indole alkaloids are also reported from this plant [8–13]. Previously, we isolated non-alkaloid constituents [14] as well as indole alkaloids [15] from *V. major* cultivated in Kunming. In our ongoing search for alkaloids from this plant growing in different habitats, we carried out the phytochemical investigation of the aerial parts of *V. major* in Pakistan, and then isolated three new pyridine alkaloids, named as (–)-vincapyridines A–C (**1**–**3**) trivially (Fig. 1). The structures of new alkaloids were elucidated by means of spectroscopic methods. The new alkaloids were evaluated for their cytotoxicity against human glioma initiating cell lines (GITC-3^#^ and GITC-18^#^), glioblastoma cell lines (U-87MG and T98G), and lung cancer cell line A-549 using the reported MTS assay with DMSO as the control group. Unfortunately, none of these compounds exhibited significant cytotoxicity at 20 μg/mL concentration. Herein, we report the isolation, structural elucidation of these compounds.

## Results and Discussion

**Fig. 2:**
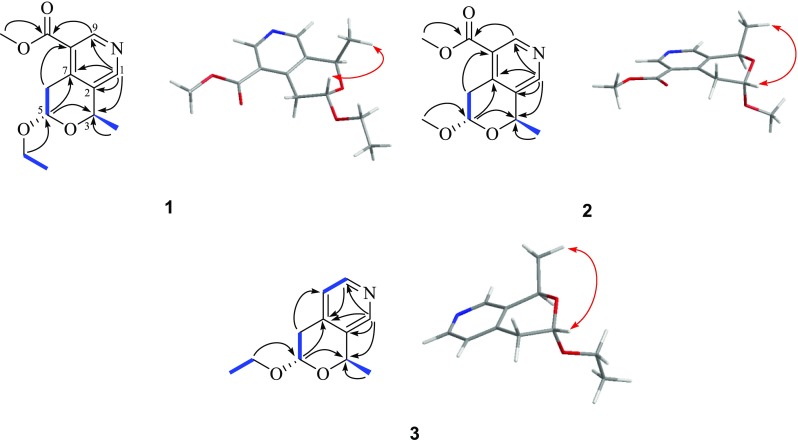
Selective HMBC (→), ^1^H–^1^H COSY (−), and ROESY (↔) correlations for **1**–**3**

The molecular formula of **1** was determined to be C_13_H_17_NO_4_, by a quasi-molecular ion peak at *m/z* 252.1222 [M+H]^+^ (calcd for C_13_H_18_NO_4_, 252.1230) in the positive HRESIMS. Its IR spectrum revealed a characteristic absorption band at 1724 (C=O). The ^1^H and ^13^C NMR spectral data of **1** (Table 1), assumed that **1** was a tri-substituents pyridine derivative with two typical downfield protons [*δ*_H_ 8.89 (s) and 8.53 (s)] assigned to be pyridine aromatic protons [16]. In HMBC spectrum of **1**, correlations of pyridine protons *δ*_C_ 8.53 (s) with substituents aromatic carbons at *δ*_C_ 146.3 (C-7), 137.6 (C-2), and oxymethine at *δ*_C_ 71.3 (C-3), indicated substituents pattern (Fig. 2). Moreover, HMBC correlations of *δ*_H_ 4.88 (H-5) with *δ*_C_ 71.3 (C-3) and *δ*_C_ 146.3 (C-7) established a substituents pyranose ring fused to the pyridine ring (Fig. 2). Besides, HMBC correlations of *δ*_H_ 1.64 (3H, d, –CH_3_) with *δ*_C_ 71.3 (C-3), and of *δ*_H_ 3.99 and 3.62 (2H, –OCH_2_CH_3_) with *δ*_C_ 99.6 (C-5), placed a methyl at C-6 and oxyethyl group at C-5, respectively, which was further supported by the correlations of *δ*_H_ 3.37/4.88, 3.99/1.24, and 5.02/1.64, in its ^1^H–^1^H COSY spectrum (Fig. 2). Finally, the HMBC correlations of *δ*_H_ 8.89 (s) and 3.92 (–OCH_3_) with *δ*_C_ 167.2 (–COOCH_3_) positioned the methyl formate group at C-8. In ROESY spectrum of **1**, NOE correlation between *δ*_H_ 4.88 (H-5) and 1.64 (H-4) revealed two protons to be co-facial (Fig. 2), which indicated its relative configuration.

**Table 1:** ^1^H and ^13^C NMR spectroscopic data of **1**–**3** (*δ* in ppm, *J* in Hz)

Position	**1**		**2**		**3**	
	*δ*_H_^a^	*δ*_C_^b^	*δ*_H_^a^	*δ*_C_^b^	*δ*_H_^a^	*δ*_C_^b^
1	8.53 (s)	149.8	8.51 (s)	149.5	8.33 (br. s)	146.0
2		137.6		137.5		137.2
3	5.02 (q, 6.4)	71.5	4.98 (q, 6.6)	64.5	4.94 (q, 6.6)	64.7
4	1.64 (d, 6.4)	21.9	1.58 (d, 6.6)	21.4	1.57 (d, 6.6)	21.0
5	4.88 (dd, 3.2, 8.2)	99.6	5.07 (dd, 1.2, 4.6)	97.7	5.17 (dd, 1.4, 4.6)	96.7
6	3.37 (dd, 3.2, 18.2) 3.07 (dd, 8.2, 18.2)	35.0	3.35 (dd, 4.6, 18.6) 3.21 (br. d, 18.6)	33.2	3.14 (dd, 4.6, 17.3) 2.75 (d, 17.3)	34.3
7		146.3		144.7		143.5
8		126.9		126.6	7.18 (d, 5.2)	125.5
9	8.89 (s)	150.2	8.88 (s)	150.1	8.29 (br. d, 5.2)	147.8
–OCH_2_CH_3_	3.99 (dq, 7.1, 9.4) 3.62 (dq, 7.1, 9.4)	65.4			3.85 (dq, 7.1, 9.7) 3.58 (dq, 7.1, 9.7)	64.3
–OCH_2_ CH_3_	1.24 (t, 7.1)	15.7			1.19 (t, 7.1)	15.5
–COOCH_3_		167.2		167.3		
–COOCH_3_	3.92 (s)	52.9	3.92 (s)	52.9		
–OCH_3_			3.44 (s)	55.6		

^a^Recorded at 600 MHz in CD_3_OD

^b^Recorded at 150 MHz in CD_3_OD

The molecular formula of **2** was established as C_12_H_15_NO_4_ by a quasi-molecular ion peak in the HRESIMS at *m*/*z* 238.1072 [M+H]^+^ (calcd for C_12_H_16_NO_4_, 238.1074). The ^1^H and ^13^C NMR spectral data of **2** were similar to those of **1**, except for a methoxyl group connected to C-5 in **2** instead of the ethoxyl group in **1**, which was consistent with its molecular formula, and further supported by HMBC correlation. Compound **2** shared same relative configurations with **1**, supported by NOE correlation between H-5 and H-4 (Fig. 2).

Its molecular formula of **3** was deduced to be C_11_H_15_NO_2_ on the basis of HRESIMS quasi-molecular ion peak at *m/z* 194.1172 [M+H]^+^ (calcd for C_11_H_16_NO_2_, 194.1176) and ^13^C NMR data (Table 1). Signals for methyl formate group at C-8 in the ^1^H and ^13^C NMR spectra of **1** were absent in those of **3**,and corresponding *δ*_H_ 7.18 (d, 5.2, H-8) and *δ*_C_ 125.5 (d, C-8) were appeared in the ^1^H and ^13^C NMR spectra of **3**. Other parts of **3** were identical to those of **1**, supported by its HMBC and ROSEY spectra (Fig. 2).

**Fig. 3:**
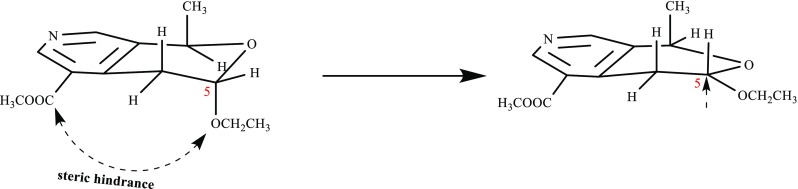
Conformation change of compound **1** indicated by molecular model

The optical rotation of **1**–**3** with same negative sign, supposed their same absolute configuration. However, the specific rotation values of **1**–**3** were obviously different, which indicated that substituents at C-8 and 5 contributed to specific rotation significantly. In compound **1**, to avoid steric hindrance between –COOCH_3_ and –OCH_2_CH_3_ (Fig. 3), the dihedral angle between H-6 and H-5 were changed, resulting in a large coupling constants (*J* = 8.4 Hz) for H-6/H-5 in its ^1^H NMR spectrum, which were different from those of two other compounds. Meanwhile, the deformed substituent pyran forward the hemiacetal proton (H-5) to the shield area of pyridine ring (Fig. 3), which caused the up-field chemical shift of H-5 (*δ*_H_ 4.88) in **1,** comparison of those in **2** (*δ*_H_ 5.07) and **3** (*δ*_H_ 5.17). It is the first report of pyridine type alkaloids from genus *Vinca*. Besides, known indole alkaloids nauclefine (**4**) [17, 18] and vincamajoreine (**5**) [19] were also isolated.

## Experimental Section

### Plant Material

The aerial parts of *V. major* were collected from Oghi, Mansehra, KPK, Pakistan in June, 2015 and identified by one of us (A. Khan). A voucher specimen (No. Khan_20150601) has been deposited in the State Key Laboratory of Phytochemistry and Plant Resources in West China, Kunming Institute of Botany, Chinese Academy of Sciences, China.

### General Experimental Procedures

Optical rotations were performed on a P-1020 polarimeter. IR spectra were measured on a Bruker FT-IR Tensor 27 spectrometer with KBr pellets. UV spectra were obtained on Shimadzu UV-2401A spectrometer. 1D and 2D-NMR spectra were recorded on Bruker AV-600 MHz spectrometer. Coupling constants were expressed in Hertz and chemical shifts were given on a ppm scale with tetramethylsilane as an internal standard. HRESIMS were recorded on an API QSTAR Pulsar 1 spectrometer. Column chromatography (CC) was performed on silica gel (200–300 mesh, Qingdao Marine Chemical Ltd., Qingdao, People’s Republic of China), Sephadex LH-20 (Pharmacia Fine Chemical Co., Ltd., Sweden), and MCI-gel CHP 20P (75–100 μm, Mitsubishi Chemical Co., Ltd). Thin-layer chromatography (TLC) was carried out on pre-coated silica gel plates (Qingdao Marine Chemical Co., Ltd.) with CHCl_3_/MeOH (15:1, 4:1, v/v) as developing solvents and spots were visualized by Dragendorff’s reagent. High performance liquid chromatography (HPLC) was performed using Waters 600 pump with semi-preparative C_18_ columns (150 × 9.4).

### Extraction and Isolation

The air-dried and powdered aerial parts of *V. major* (8 kg) were extracted with 80% aqueous MeOH (80 L × 3) at room temperature. After removal of the organic solvent under reduced pressure, the residue was dissolved in 0.3% aqueous hydrochloric acid (v/v). The solution was subsequently basified to pH 9–10 using aqueous ammonia, and then extracted with EtOAc (3 L × 4) to give an alkaloidal extract (34.3 g). The extract was applied to a silica gel column (CHCl_3_/MeOH, 1:0–0:1) to afford nine fractions (Fr. A-I). Fr. B (2.5 g) was subjected to silica gel column chromatography (CC) using a petroleum ether/acetone gradient eluent (10:1–9:1) to afford sub-fractions (Fr. 1–5). Fr. 4 (200 mg) was further purified on MCI-gel CHP 20P column using a MeOH/H_2_O gradient eluent (1:4–1:0) and on a semi-preparative C_18_ HPLC column with a gradient of MeOH/H_2_O (50:50–90:10) to yield vincamajoreine (**5**) (6 mg). Fr. H (5 g) was subjected to silica gel CC (CHCl_3_/MeOH, 9:1–0:1) to afford sub-fractions (Fr. 5–10). Fr. 5 (1200 mg) was further separated on MCI-gel CHP 20P column to yield nauclefine (**4**) (15 mg). Fr. 8 (800 mg) was subjected to Sephadex LH-20 CC using MeOH under isocratic conditions and was further separated on a semi-preparative C_18_ HPLC column with a gradient of MeOH/H_2_O (30:70–70:30) to produce (−)-vincapyridine A (**1**) (3 mg), (−)-vincapyridine B (**2**) (2 mg), and (−)-vincapyridine C (**3**) (1.2 mg).

(−)-vincapyridine A (**1**): C_13_H_17_NO_4_. white amorphous powder; $\left[ {\upalpha } \right]_{\text{D}}^{26}$ −26.6 (*c* 0.07, MeOH); UV (MeOH) *λ*_max_ (log ε): 203 (4.22), 220 (3.92), 270 (3.38); IR (KBr) *ν*_max_ 3418, 2929, 1724, 1572, 1381, 1295, 1079 cm^−1^; ^1^H and ^13^C NMR data, see Table 1; HRESIMS *m/z* 252.1222 [M+H]^+^ (calcd for C_13_H_18_NO_4_, 252.1230).

(−)-vincapyridine B (**2**): C_12_H_15_NO_4_. white amorphous powder; $\left[ {\upalpha } \right]_{\text{D}}^{26}$ −190.0 (*c* 0.04, MeOH); UV (MeOH) *λ*_max_ (log ε): 204 (4.44), 219 (4.13), 269 (3.57); IR (KBr) *ν*_max_ 3419, 2926, 1724, 1606, 1383, 1042 cm^−1^; ^1^H and ^13^C NMR data, see Table 1; HRESIMS *m*/*z* 238.1072 [M+H]^+^ (calcd for C_12_H_16_NO_4_, 238.1074).

(−)-vincapyridine C (**3**): C_11_H_15_NO_2_. white amorphous powder; $\left[ {\upalpha } \right]_{\text{D}}^{26}$ −359.8 (*c* 0.02, MeOH); UV (MeOH) *λ*_max_ (log ε): 204 (3.35), 279 (2.64); IR (KBr) *ν*_max_ 3425, 2926, 1720, 1623, 1383, 1035 cm^−1^; ^1^H and ^13^C NMR data, see Table 1; HRESIMS *m/z* 194.1172 [M+H]^+^ (calcd for C_11_H_16_NO_2_, 194.1176).

### Cytotoxicity Assay

GITC-3^#^ and GITC-18^#^ (glioma initiating cell lines) were established previously in Kunming Institute of Zoology from three different human glioblastoma multiform samples. These cell lines were cultured in serum-free medium supplemented with 1XB27 (Life 12587-010), 50 ng/mL EGF (PeproTech AF-100-15), and bFGF (PeproTech AF-100-18B). GITCs were cultured in laminin (Gibco 1725712) pre-mdish. The cells could adhere and normally grow without differentiation. Culture dishes were pre-coated with laminin for 4-6 h at 10 mg/mL concentration. The T98G, U-87MG, and A549 cell lines were purchased from the American Type Culture Collection (ATCC), the cells were cultured in DMEM basic medium supplemented with 10% FBS, 100 U/mL penicillin, and 100 μg/mL streptomycin in a humidified incubator at 37 °C and an atmosphere of 5% CO_2_. Cells were digested with TryplE express (Gibco 12604-021) for 3–5 min at 37 °C in cell incubator and centrifuged at 1000 rpm/min for 3 min.

Cell viability analysis was performed by MTS [3-(4,5-dimethylthiazol-2-yl)-5-(3-carboxymethoxyphenyl)-2-(4-sulfophenyl)-2H-tetrazolium, Promega ^#^G3581] assay [20, 21]. The cells were digested and seeded on a 96-well plate with 20000 cells/well. Each tumor cell line was exposed to the test compound dissolved in DMSO at concentrations of 20 μg/mL and kept in cell incubator for 72 h. MTS reagent was diluted 1:5 with fresh medium and mixed well. The old medium was removed and subsequently the fresh medium was added with 100 μL/well. The cells were incubated for 1 h. Absorbance was measured by Hybrid Reader (BioTek Synergy H1) at 490 nm. The cell viability was evaluated by percentage compared with DMSO as a control group.

## Supplementary Information

1D and 2D NMR spectra, HRESIMS, and UV spectra of compounds **1**–**3** are available as Supplementary Information.

## Electronic supplementary material

Below is the link to the electronic supplementary material.

Supplementary material 1 (PDF 1903 kb)
