# Supplementary material for: Three New Pyridine Alkaloids from Vinca major Cultivated in Pakistan
Source: Nat Prod Bioprospect. 2017 Jun 15;7(4):323–7. doi: 10.1007/s13659-017-0137-7 (PMC5507811; doi:10.1007/s13659-017-0137-7)
Supplement: Supplementary file 1 — Supplementary material 1 (PDF 1903 kb) [file 13659_2017_137_MOESM1_ESM.pdf]

Supplementary information

Three New Pyridine Alkaloids from *Vinca major* cultivated in Pakistan

Xin Wei[a,e,f,#], Afsar Khan[a,c,#], Da Song[b], Zhi Dai[d,e], Ya-Ping Liu[a,f], Hao-Fei Yu[a,e,f], Bei Wang[a,e,f], Pei-Feng Zhu[a,e,f], Cai-Feng Ding[a,e,f], Xu-Dong Zhao[d], Yi-Fen Wang[a,f,*], Xiao-Dong Luo[a,f,*]

[a] *State Key Laboratory of Phytochemistry and Plant Resources of West China, Kunming Institute of Botany, Chinese Academy of Sciences, Kunming 650201, P. R. China*

[b] *Yunnan College of Business Management, Kunming 650106, P. R. China*

[c] *Department of Chemistry, COMSATS Institute of Information Technology, Abbottabad 22060, Pakistan*

[d] *Key Laboratory of Animal Models and Human Disease Mechanisms, Chinese Academy of Sciences &Yunnan Province, Kunming Institute of Zoology, Kunming 650223, Yunnan, P. R. China*

[e] *University of Chinese Academy of Sciences, Beijing 100049, P. R. China*

[f] *Yunnan Key Laboratory of Natural Medicinal Chemistry, Kunming, 650201, P. R. China*

[#] *These authors contributed equally to this work*

Corresponding author: Kunming Institute of Botany, Chinese Academy of Sciences, Kunming 650201, P. R. China. E-mail: xdluo@mail.kib.ac.cn or wangyifen@mail.kib.ac.cn Phone: +86-871-6522-3177.

contents

Figure 1S. ^{1}H NMR spectrum of compound **1**

Figure 2S. ^{13}C NMR spectrum of compound **1**

Figure 3S. HSQC spectrum of compound **1**

Figure 4S. HMBC spectrum of compound **1**

Figure 5S. COSY spectrum of compound **1**

Figure 6S. ROESY spectrum of compound **1**

Figure 7S. HRESIMS spectrum of compound **1**

Figure 8S. UV spectrum of compound **1**

Figure 9S. ^{1}H NMR spectrum of compound **2**

Figure 10S. ^{13}C NMR spectrum of compound **2**

Figure 11S. HSQC spectrum of compound **2**

Figure 12S. HMBC spectrum of compound **2**

Figure 13S. COSY spectrum of compound **2**

Figure 14S. ROESY spectrum of compound **2**

Figure 15S. HRESIMS spectrum of compound **2**

Figure 16S. UV spectrum of compound **2**

Figure 17S. ^{1}H NMR spectrum of compound **3**

Figure 18S. ^{13}C NMR spectrum of compound **3**

Figure 19S. HSQC spectrum of compound **3**

Figure 20S. HMBC spectrum of compound **3**

Figure 21S. COSY spectrum of compound **3**

Figure 22S. ROESY spectrum of compound **3**

Figure 23S. HRESIMS spectrum of compound **3**

Figure 24S. UV spectrum of compound **3**

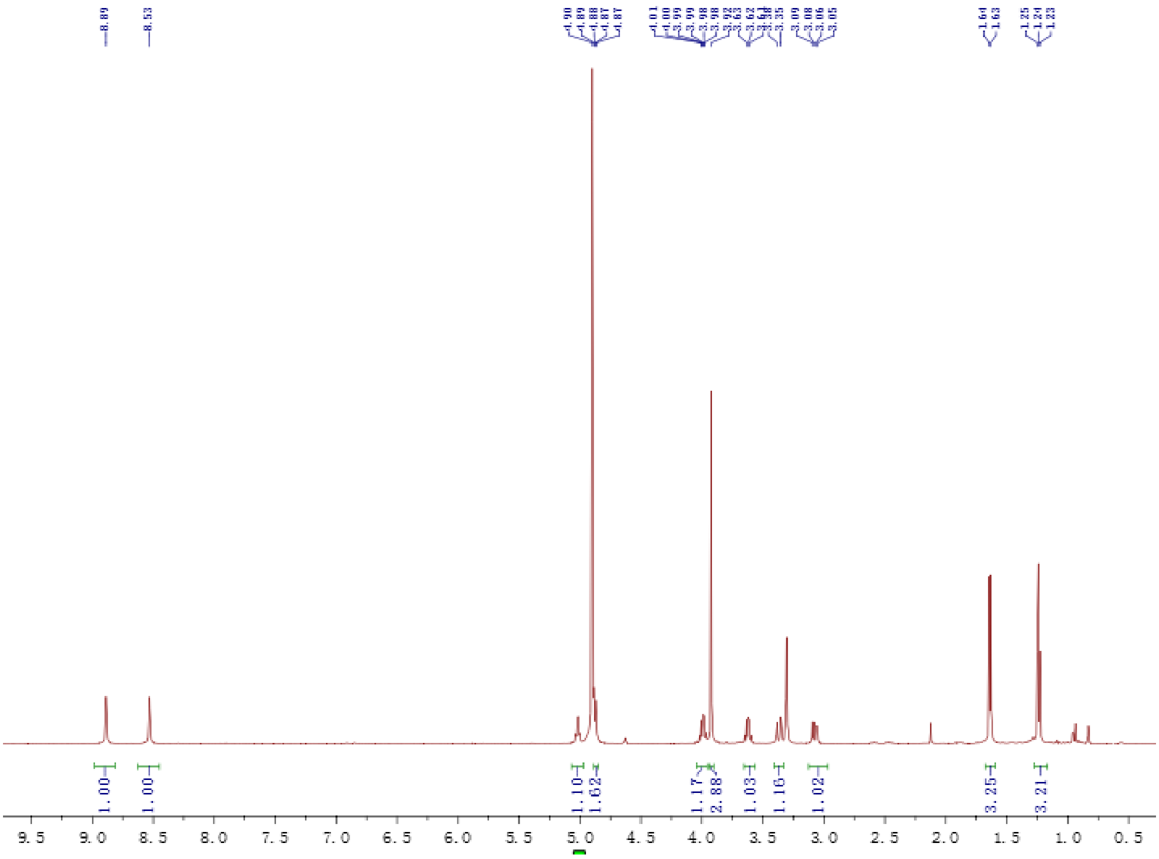

Figure 1S ^{1}H NMR spectrum of compound **1** (CD_3OD, 600 MHz)

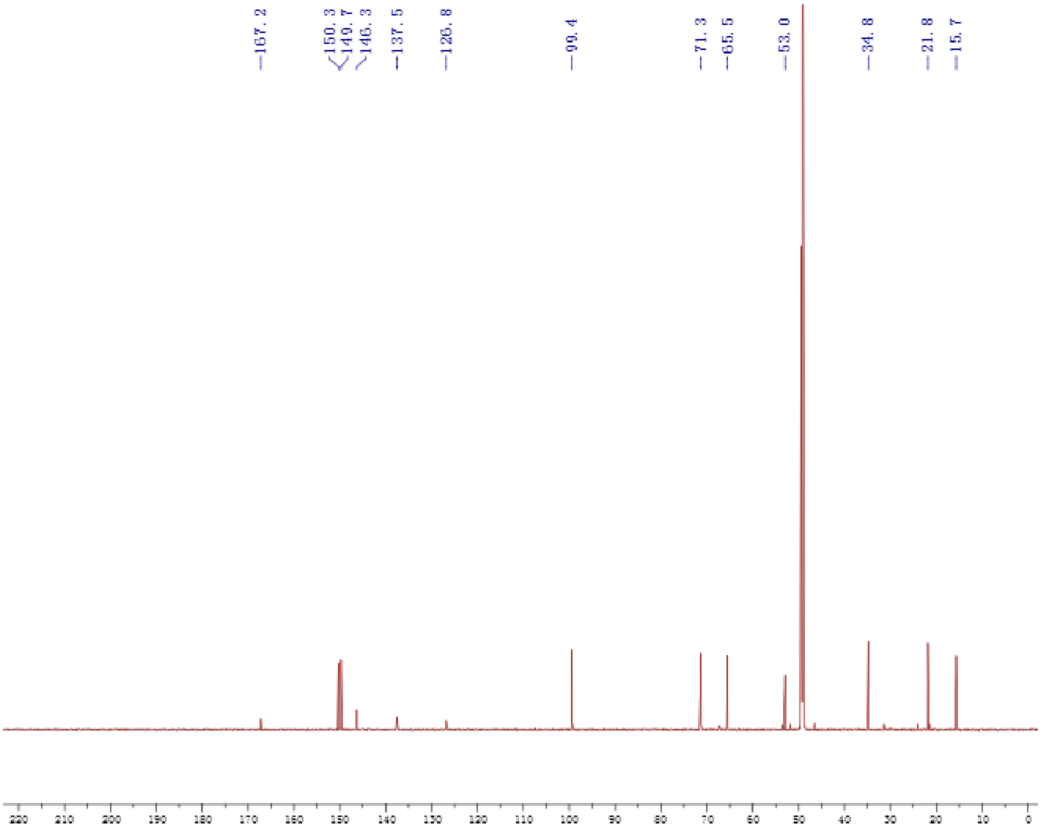

Figure. 2S ^{13}C NMR spectrum of compound **1** (CD_3OD, 150 MHz)

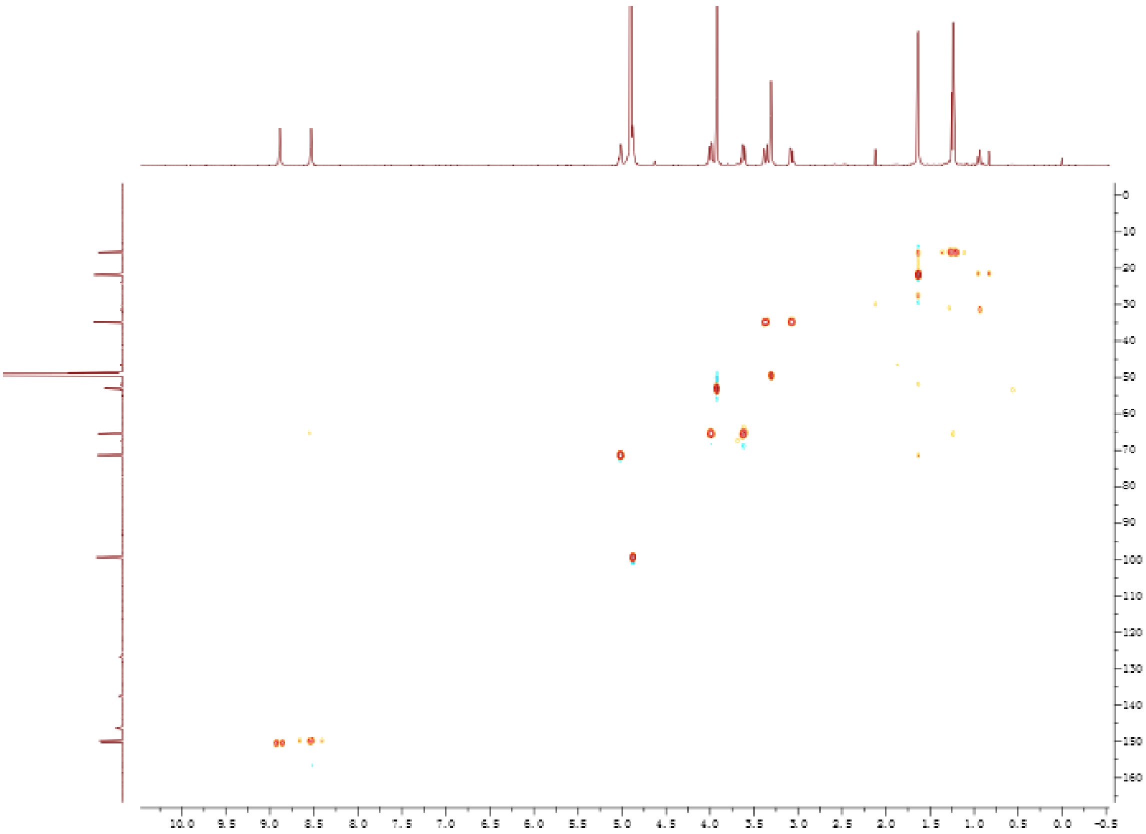

Figure. 3S HSQC spectrum of compound **1** (CD_3OD, 600 MHz)

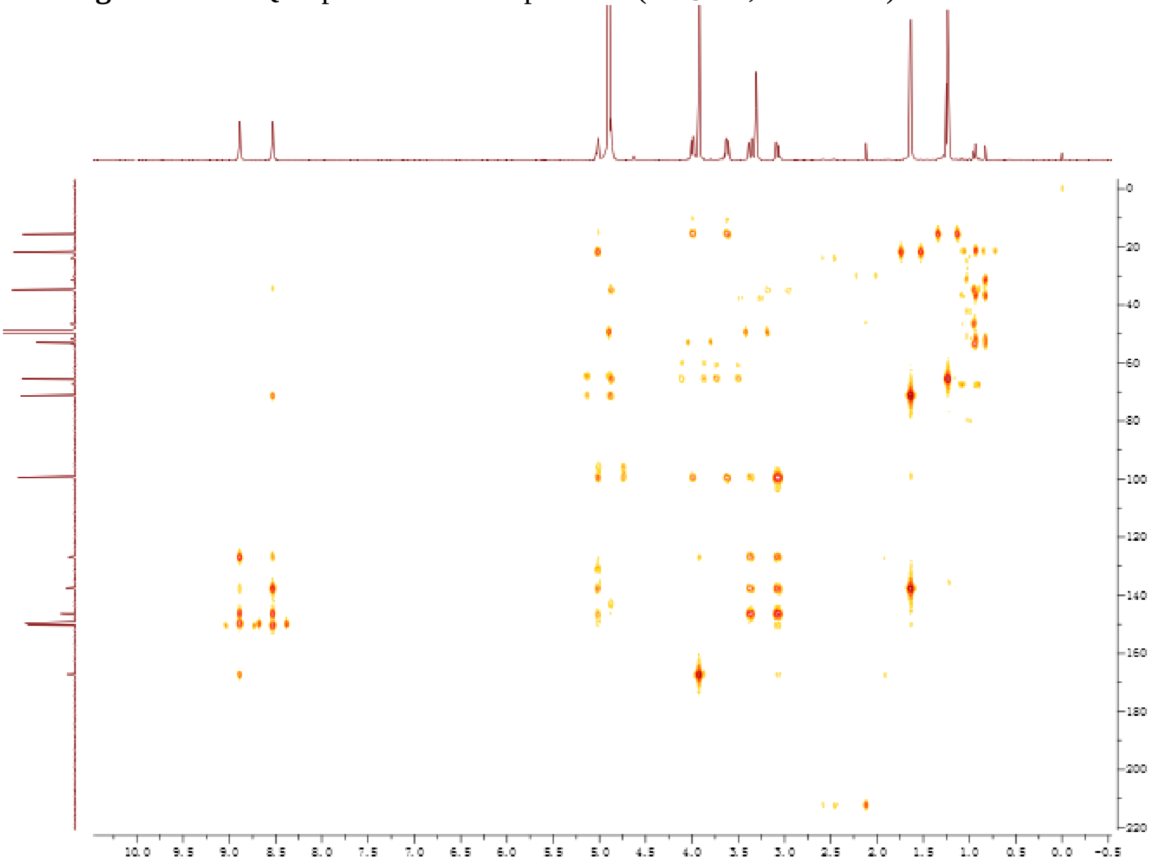

Figure. 4S HMBC spectrum of compound **1** (CD_3OD, 600 MHz)

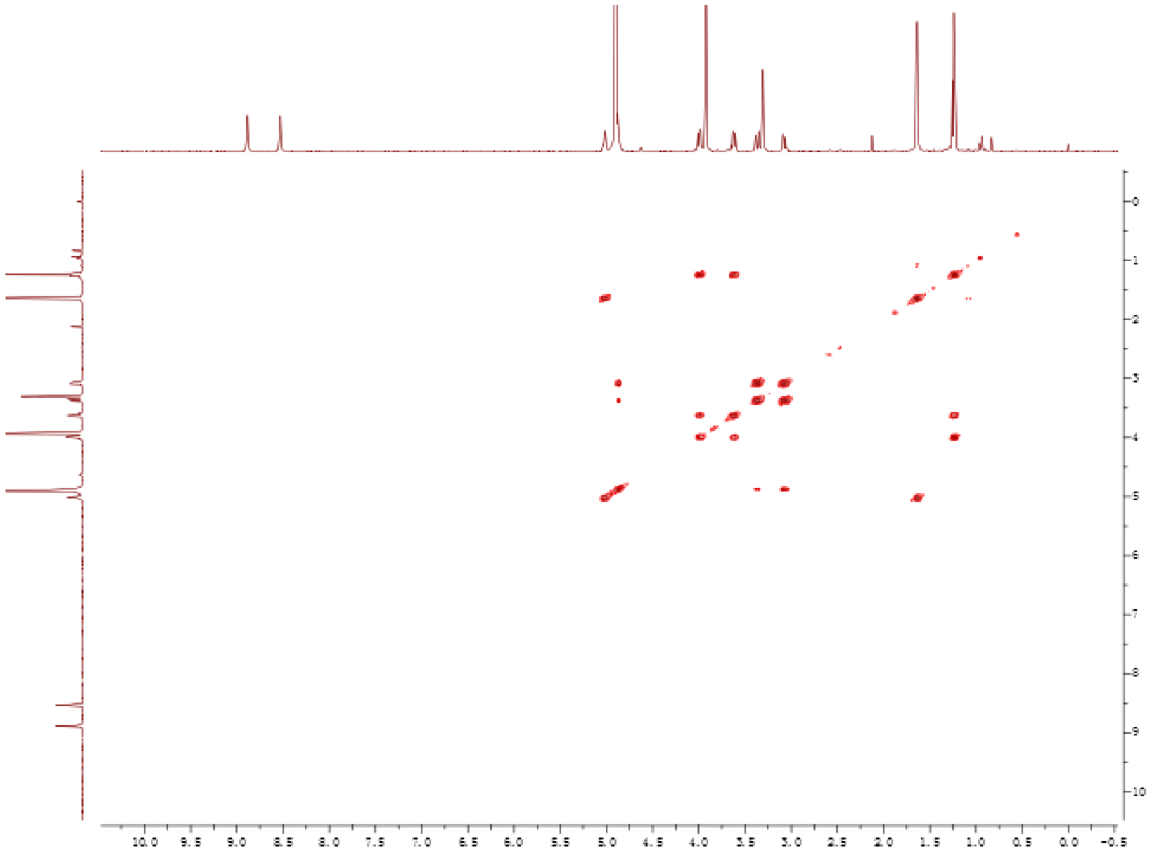

Figure. 5S COSY spectrum of compound **1** (CD_3OD, 600 MHz)

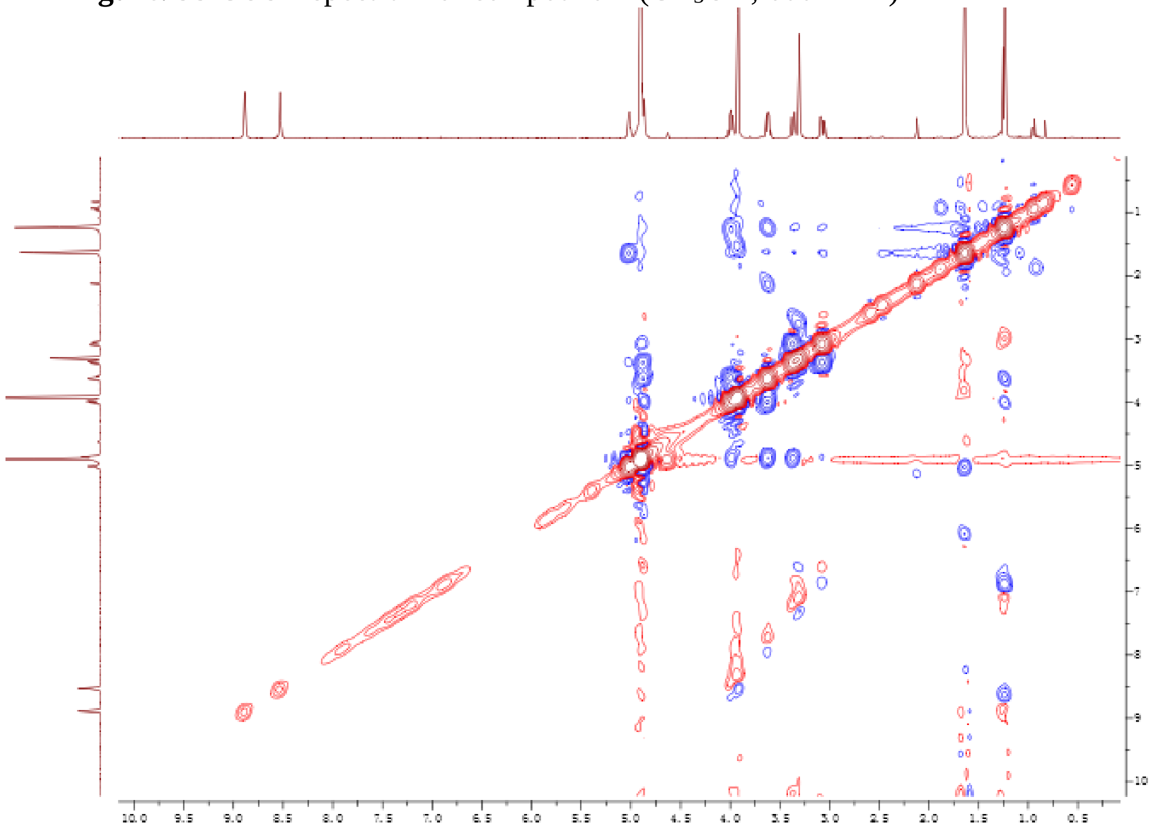

Figure 6S ROESY spectrum of compound **1** (CD_3OD, 600 MHz)

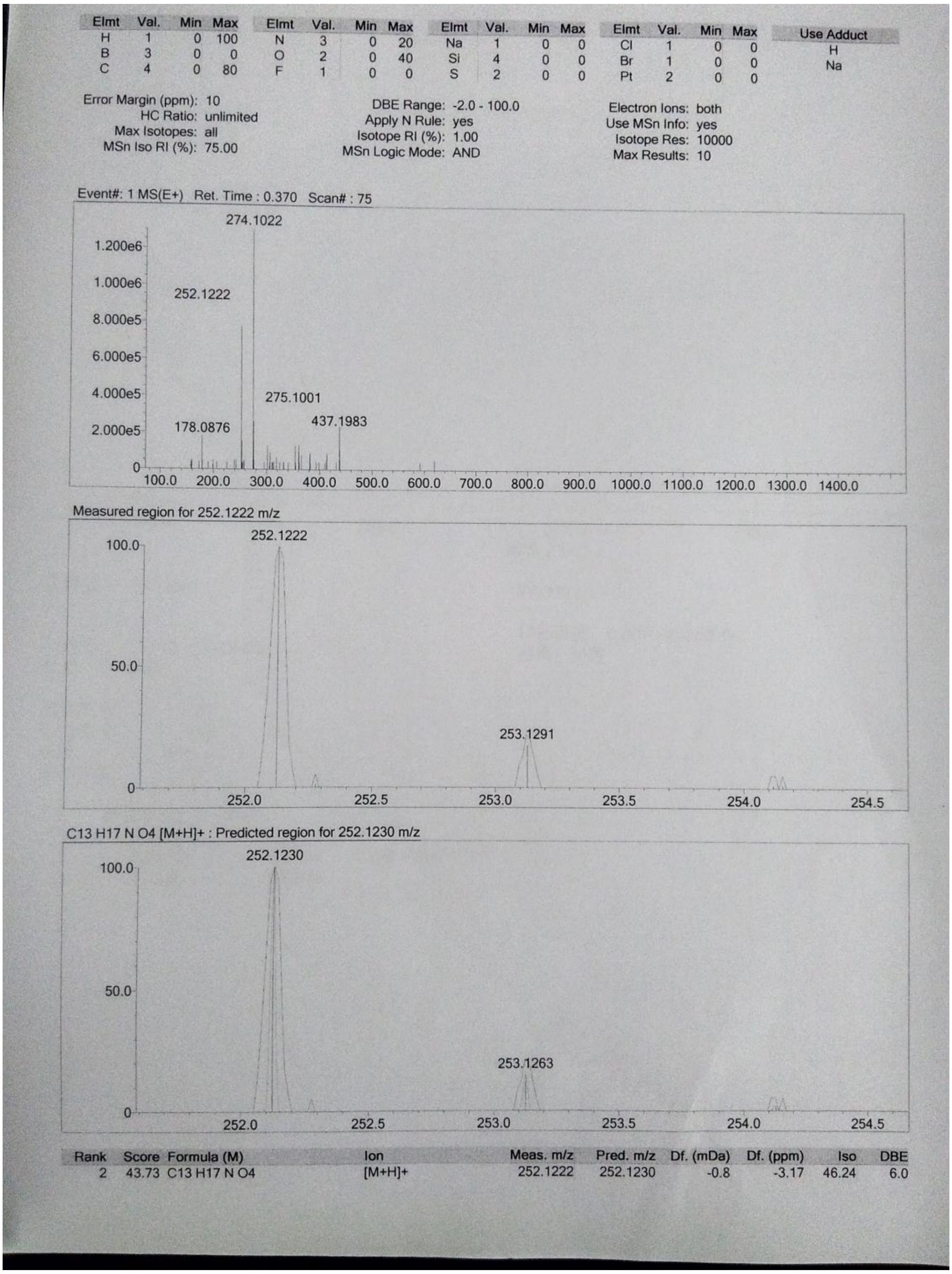

Figure 7S HRESIMS spectrum of compound **1**

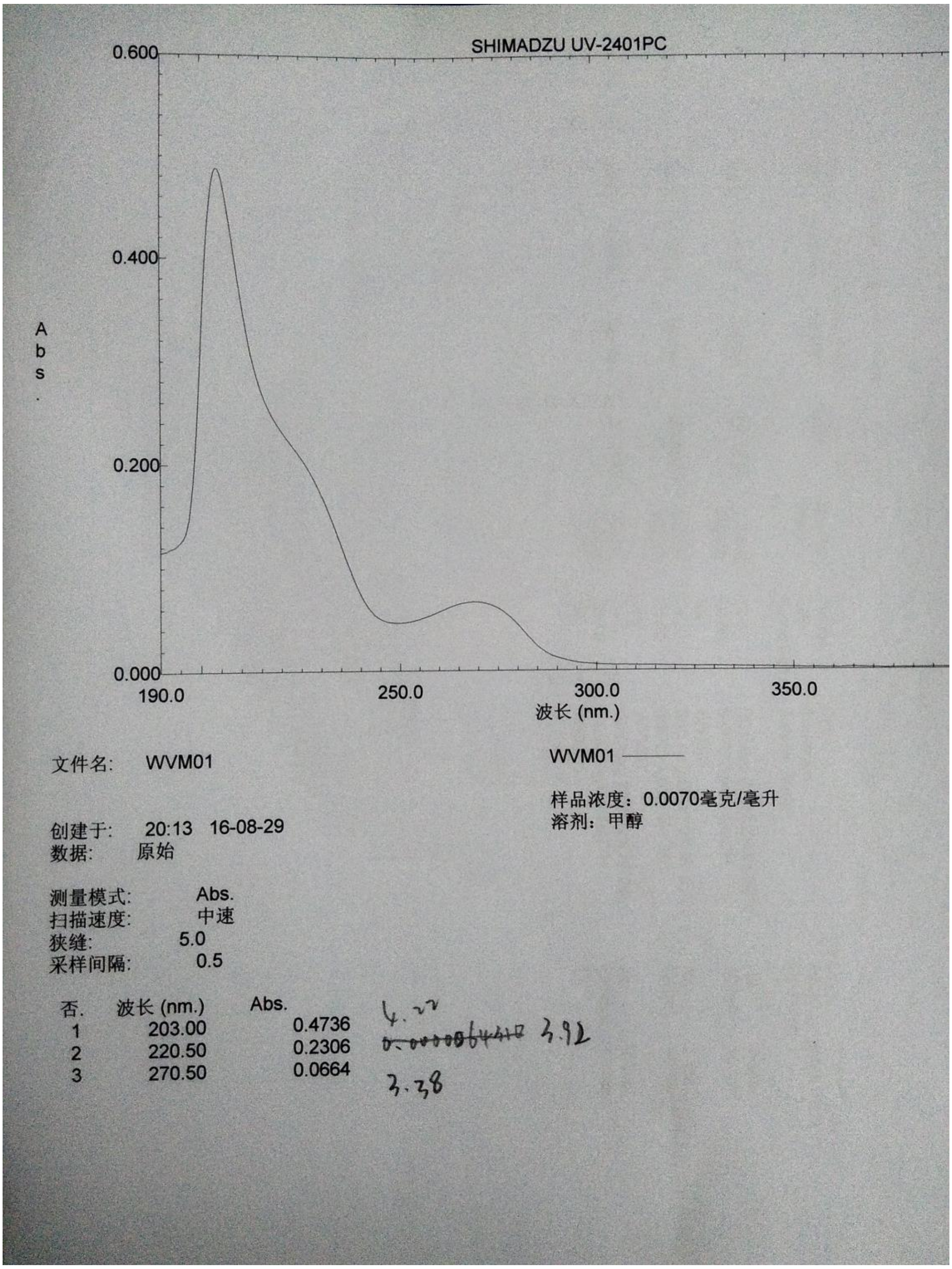

Figure 8S UV spectrum of compound **1**

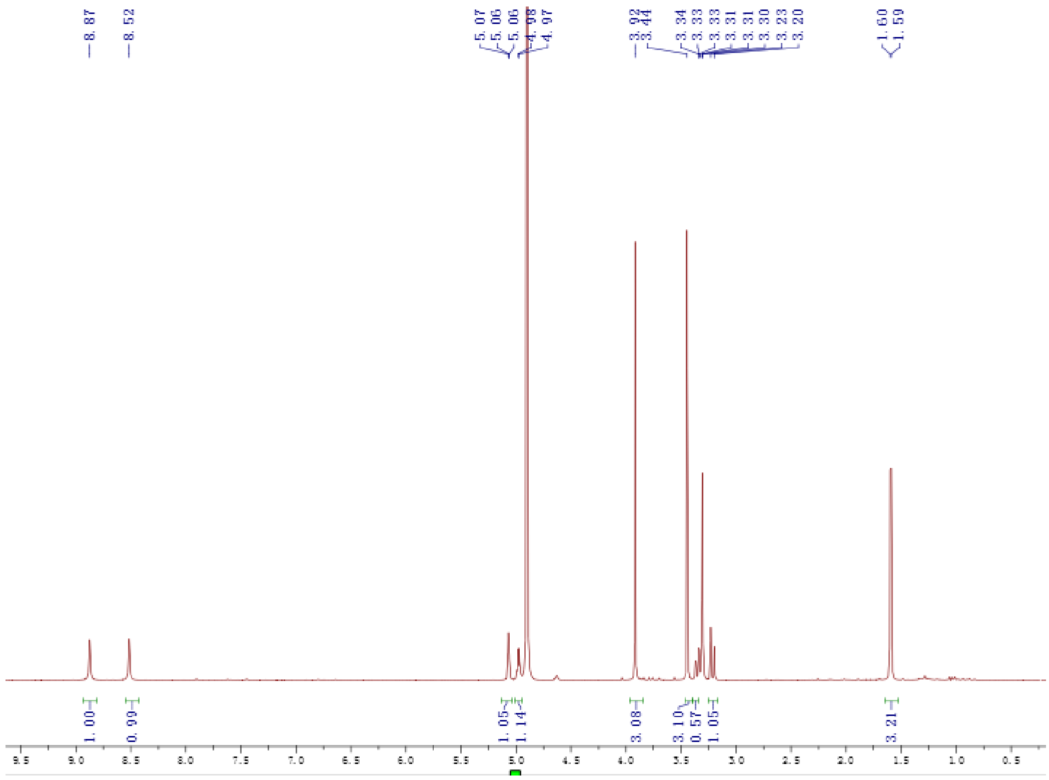

Figure. 9S ^{1}H NMR spectrum of compound **2** (CD_3OD, 600 MHz)

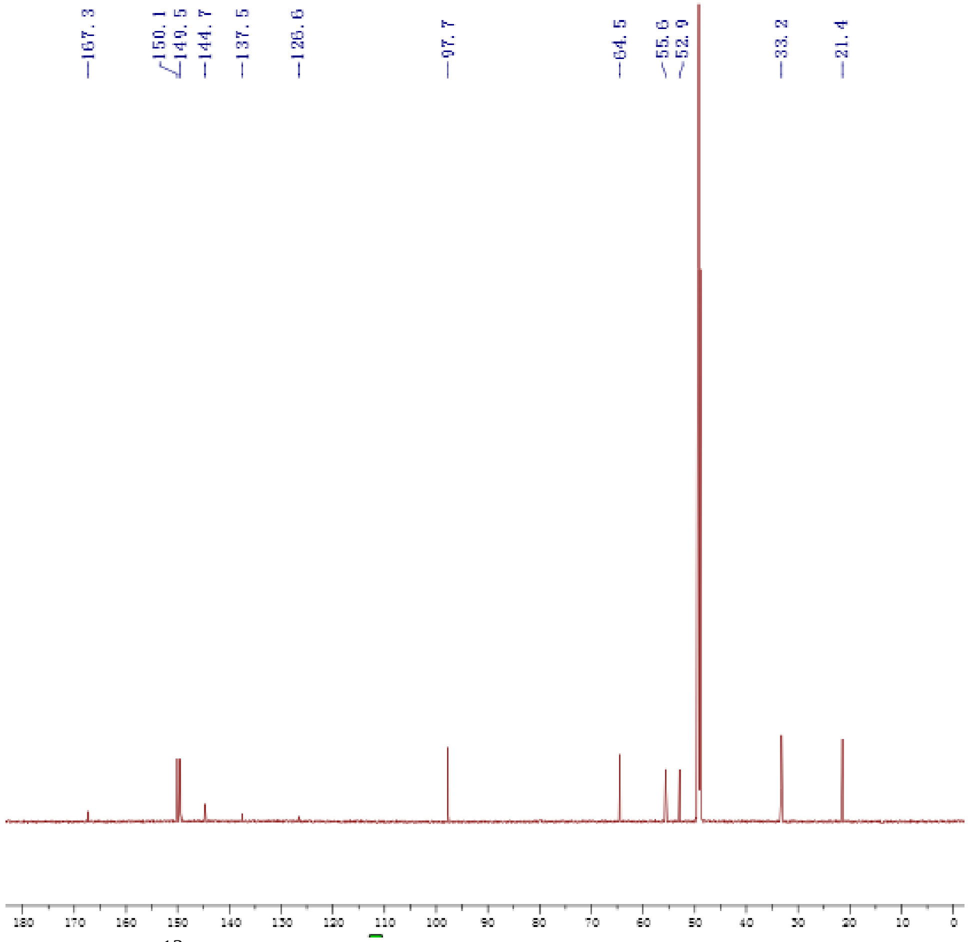

Figure 10S ^{13}C NMR spectrum of compound **2** (CD_3OD, 150 MHz)

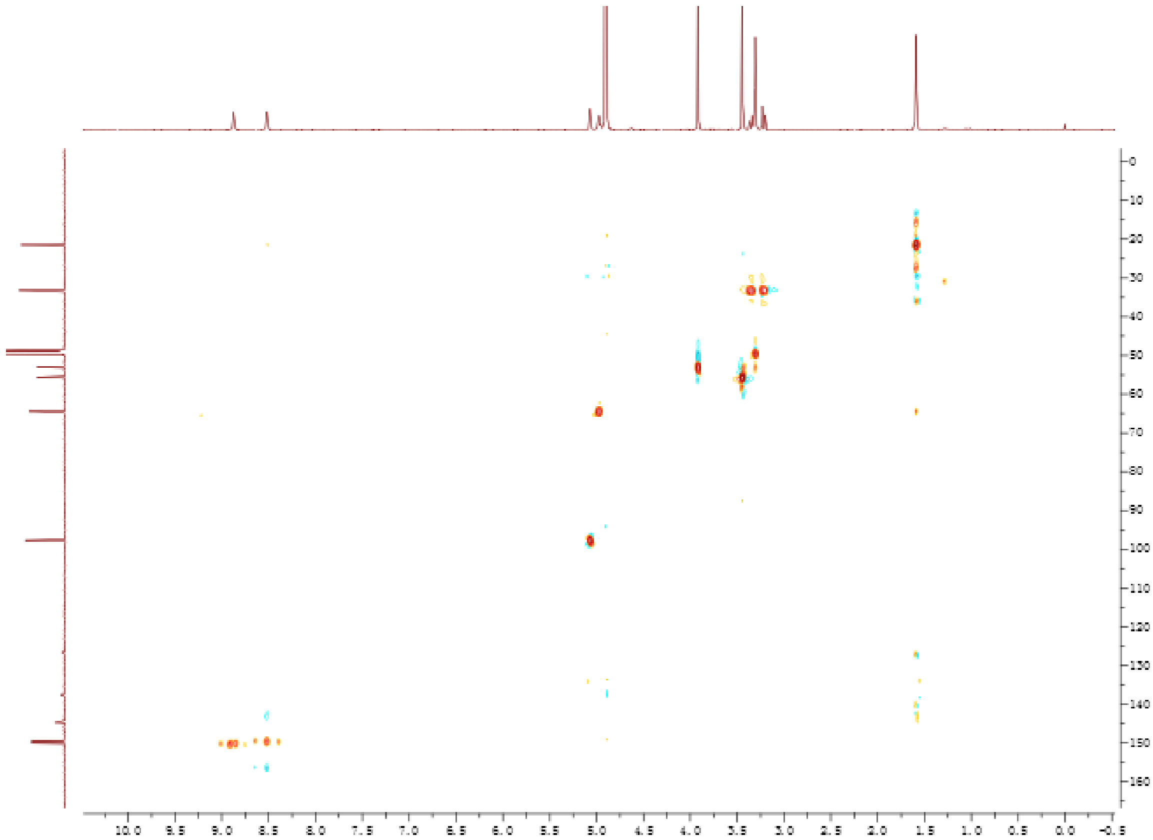

Figure 11S HSQC spectrum of compound **2** (CD_3OD, 600 MHz)

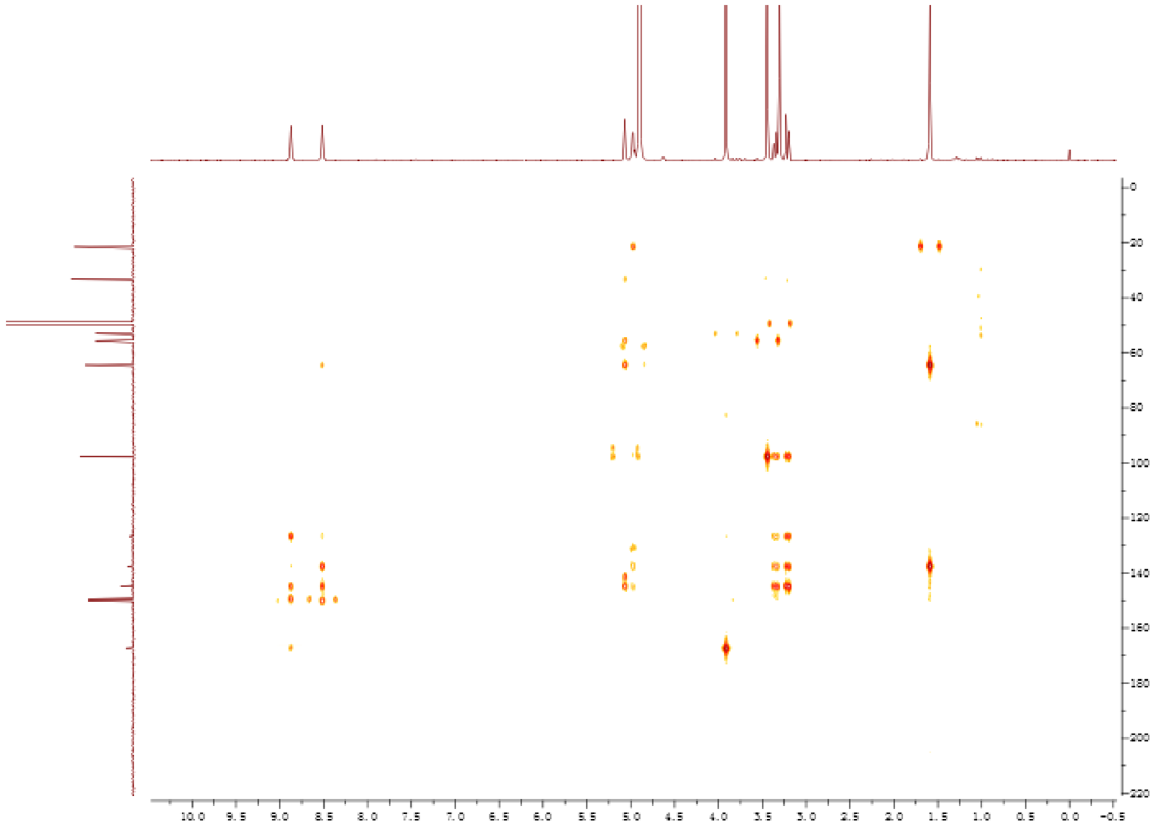

Figure 12S HMBC spectrum of compound **2** (CD_3OD, 600 MHz)

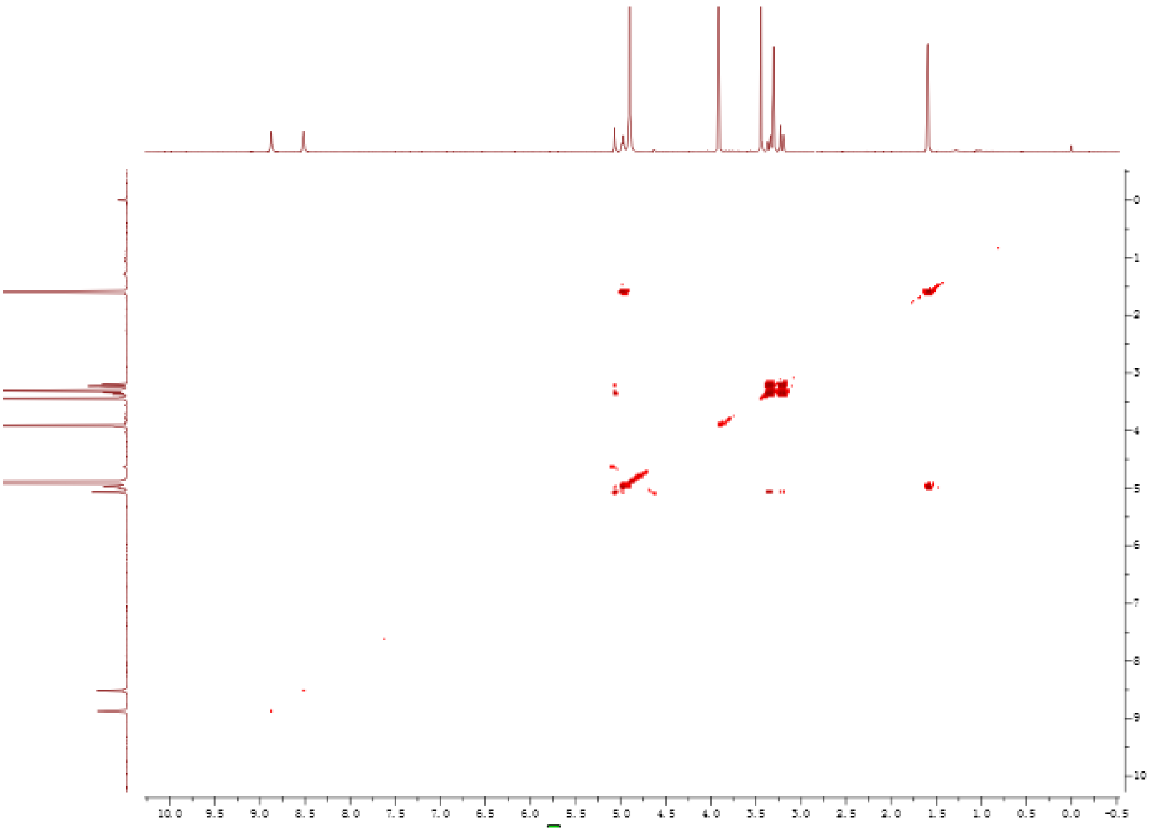

Figure 13S COSY spectrum of compound **2** (CD_3OD, 600 MHz)

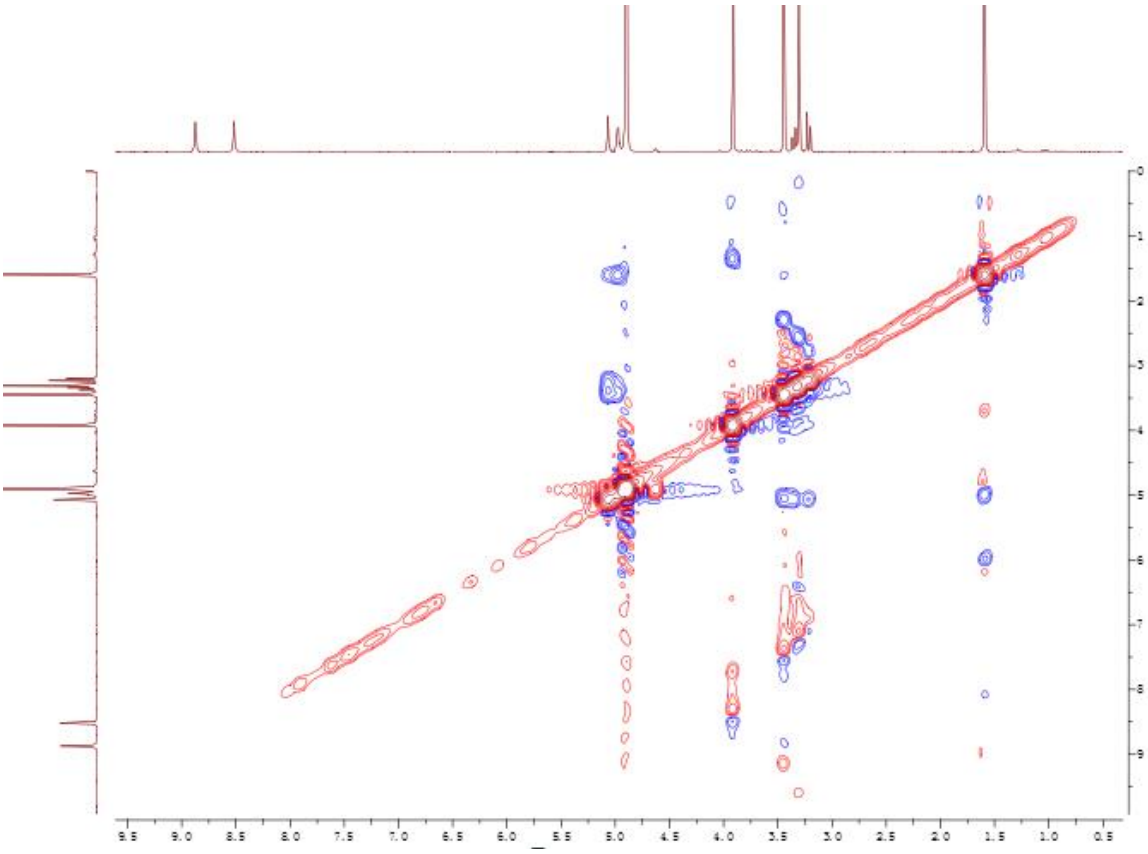

Figure 14S ROESY spectrum of compound **2** (CD_3OD, 600 MHz)

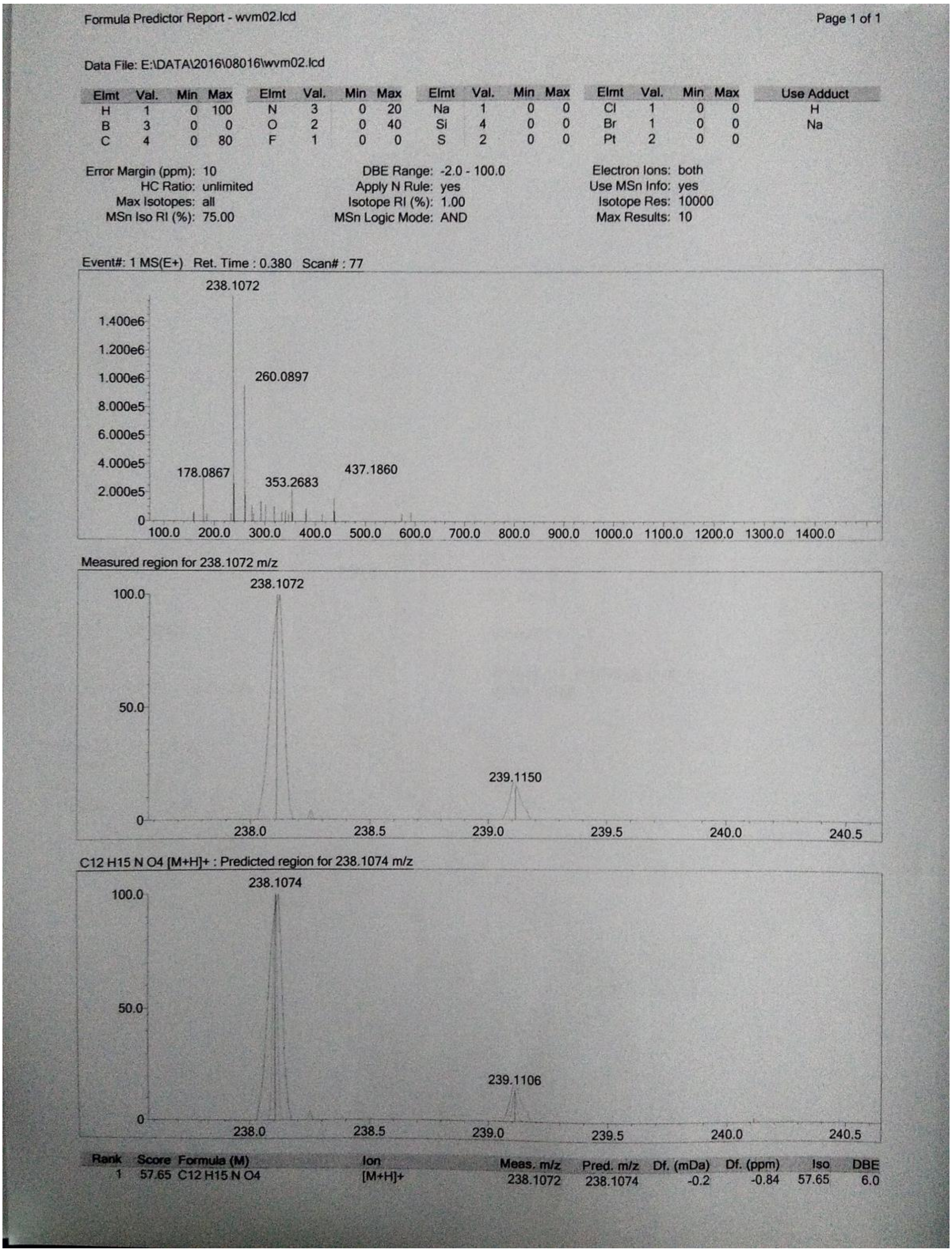

Figure 15S HRESIMS spectrum of compound **2**

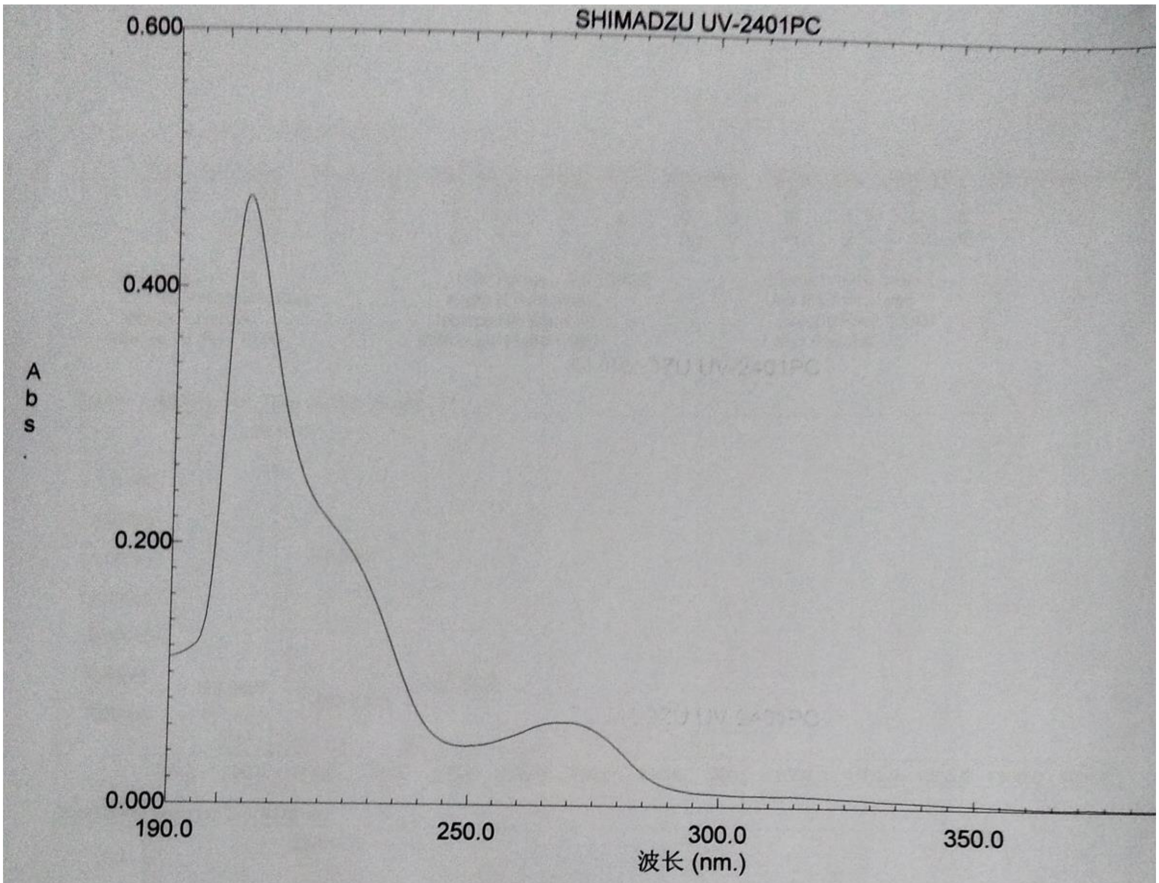

文件名: WVM02

WVM02 ———

创建于: 20:24 16-08-29
数据: 原始

样品浓度：0.0040毫克/毫升
溶剂：甲醇

测量模式: Abs.
扫描速度: 中速
狭缝: 5.0
采样间隔: 0.5

否.	波长 (nm.)	Abs.	
1	204.50	0.4711	(4.44)
2	219.00	0.2295	(4.14)
3	269.00	0.0634	(3.57)

Figure 16S UV spectrum of compound **2**

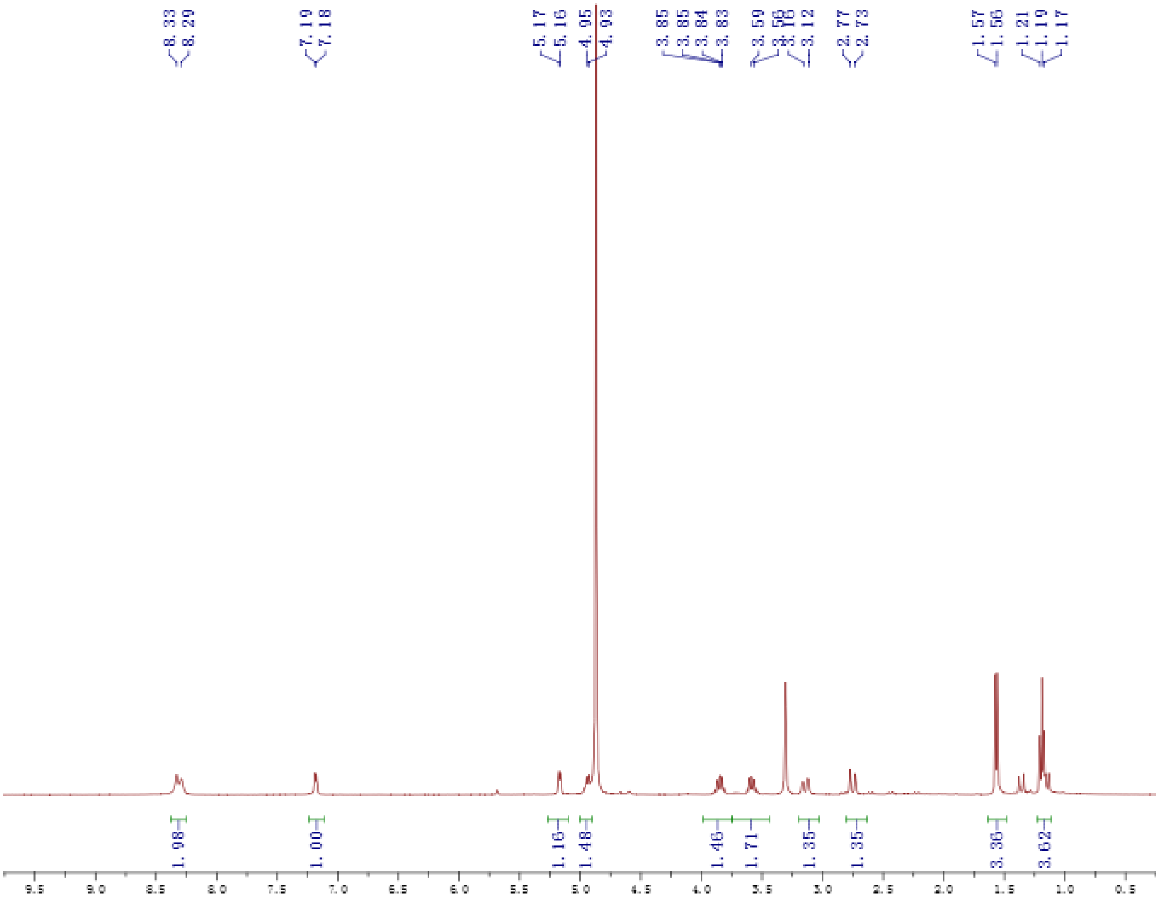

Figure 17S ^{1}H NMR spectrum of compound **3** (CD_3OD, 600 MHz)

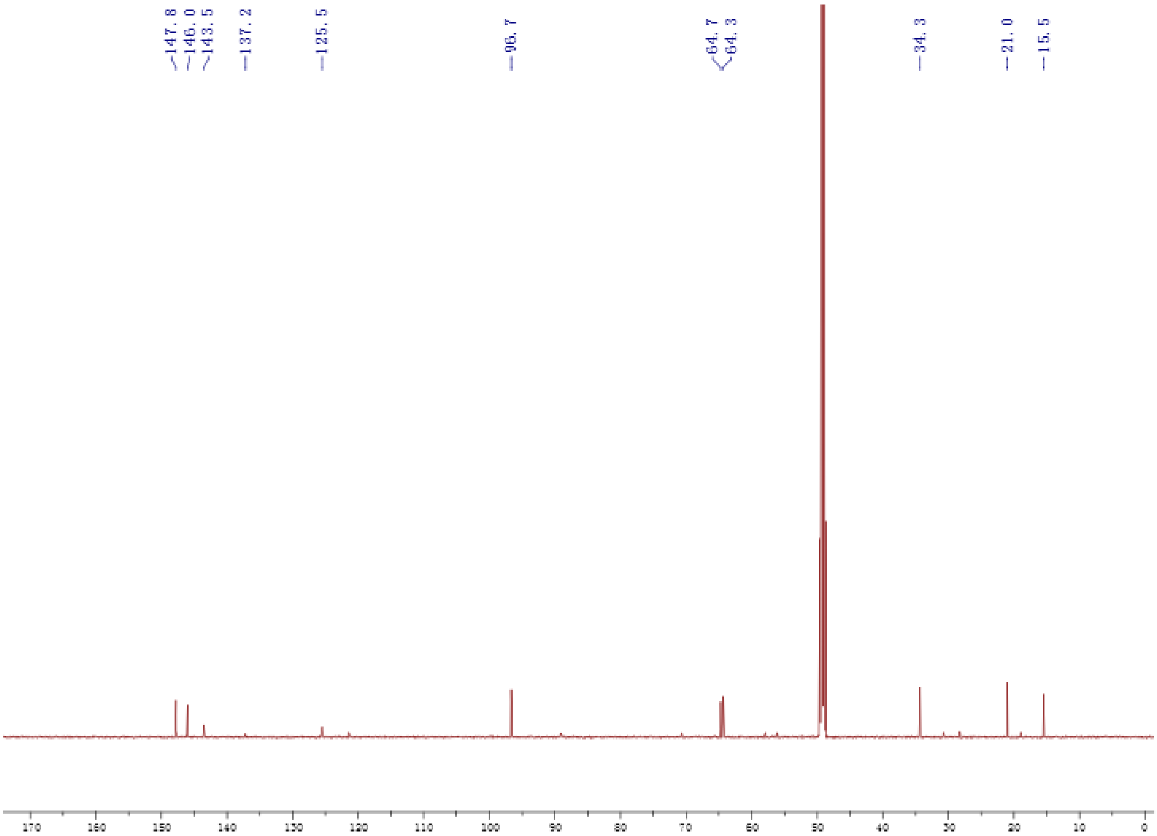

Figure 18S ^{13}C NMR spectrum of compound **3** (CD_3OD, 150 MHz)

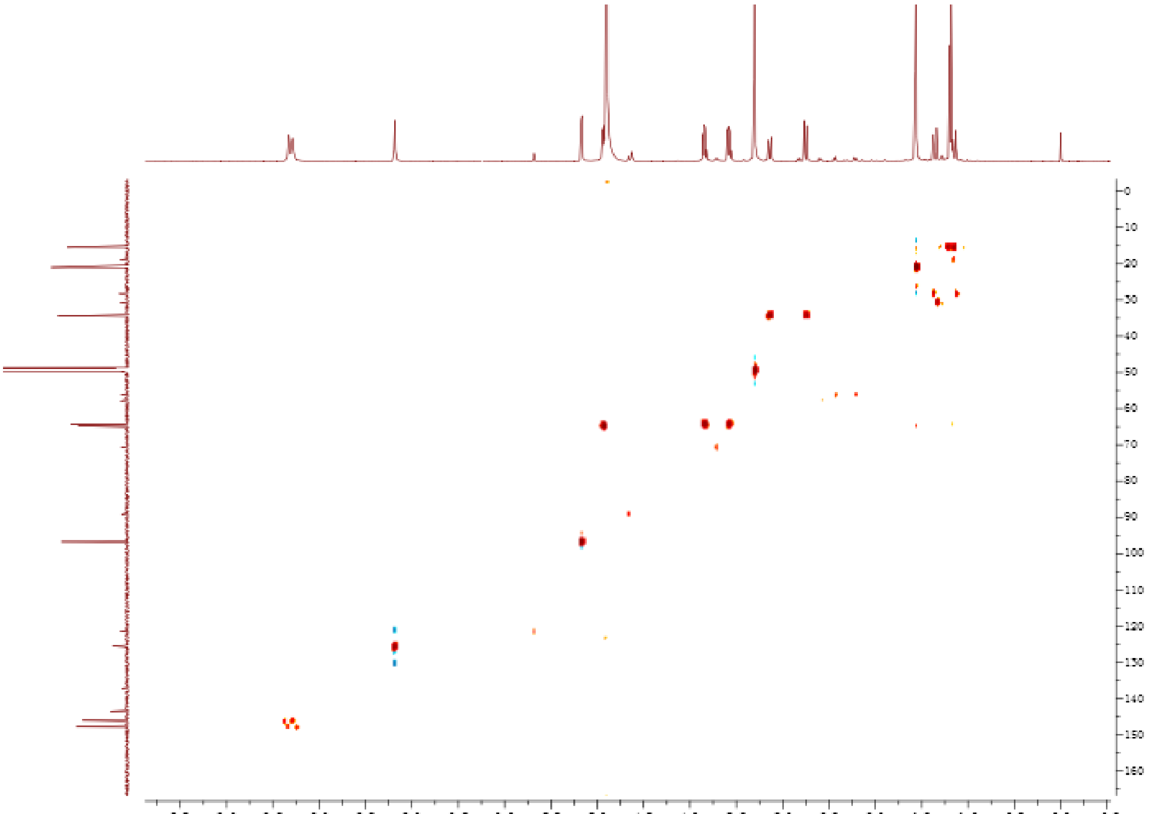

Figure 19S HSQC spectrum of compound **3** (CD_3OD, 600 MHz)\

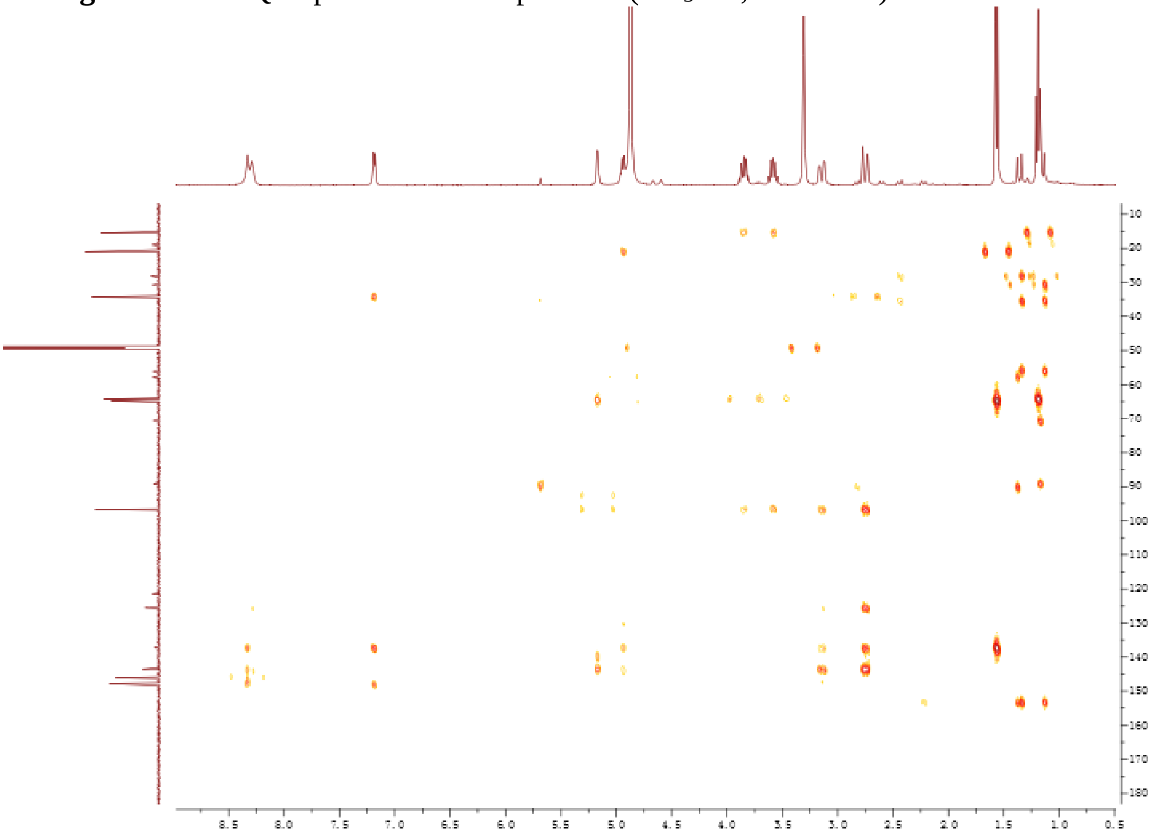

Figure 20S HMBC spectrum of compound **3** (CD_3OD, 600 MHz)

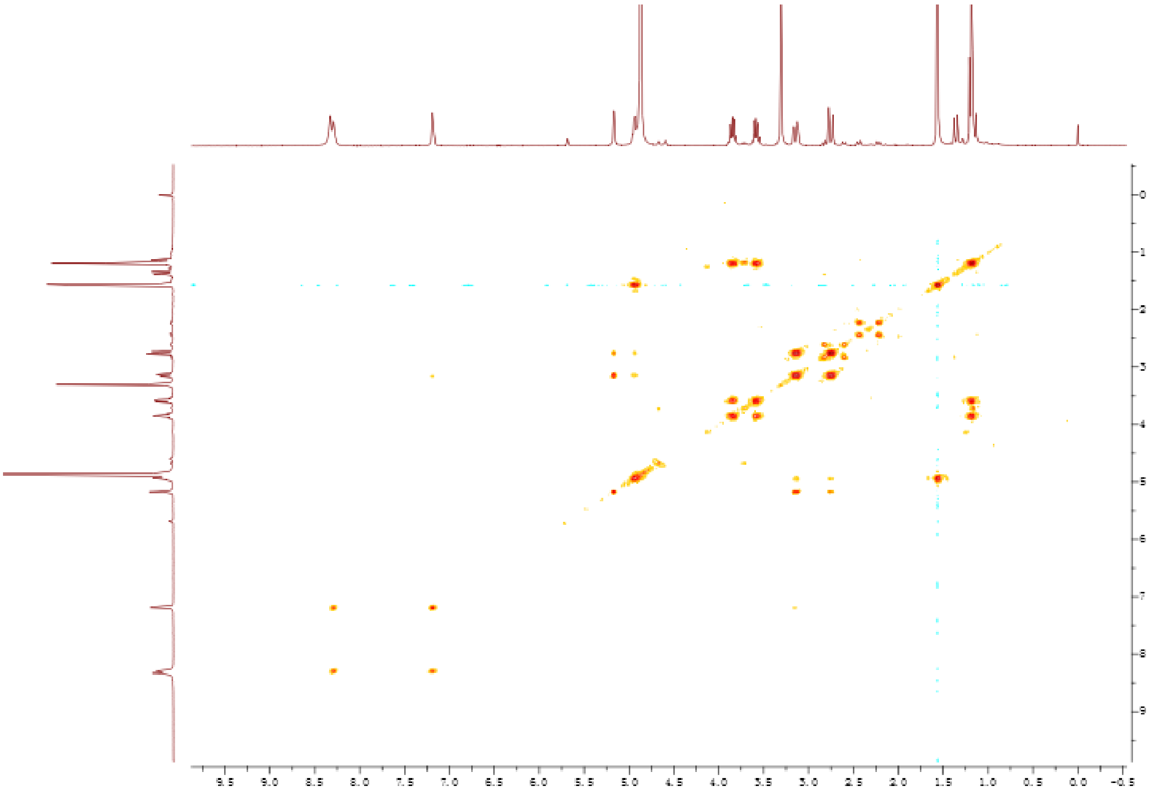

Figure 21S COSY spectrum of compound **3** (CD_3OD, 600 MHz)

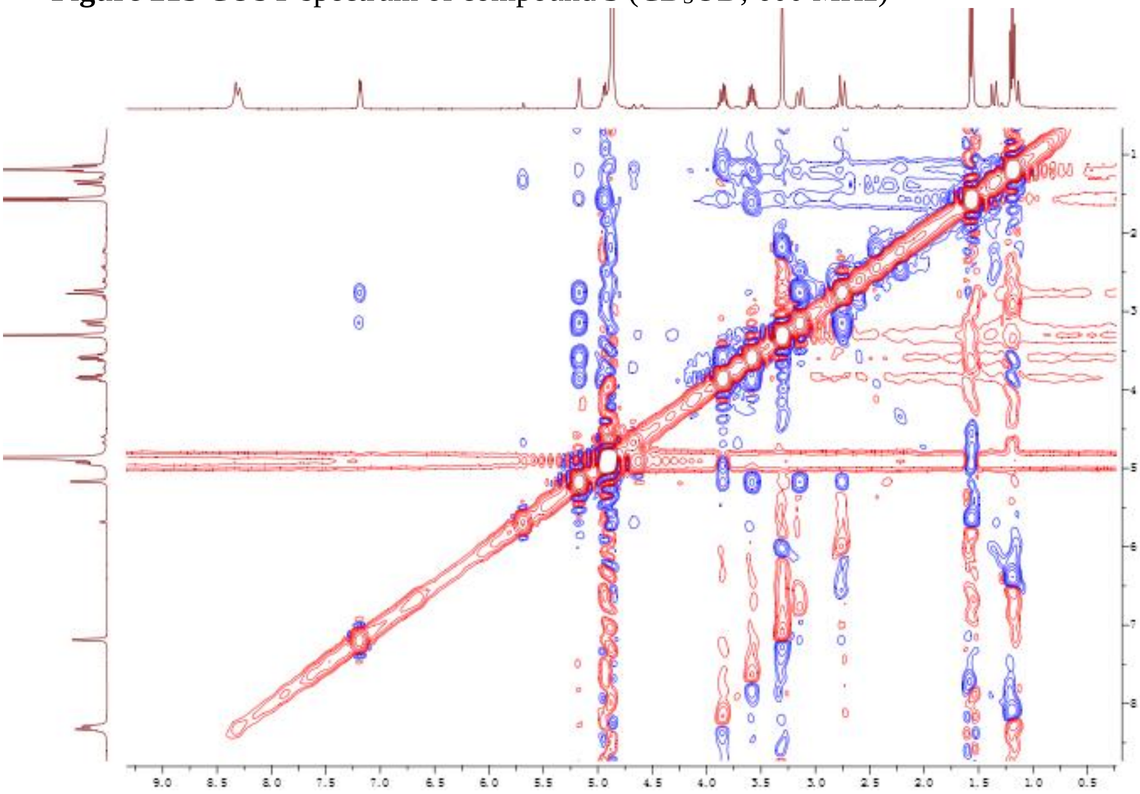

Figure 22S ROESY spectrum of compound **3** (CD_3OD, 600 MHz)

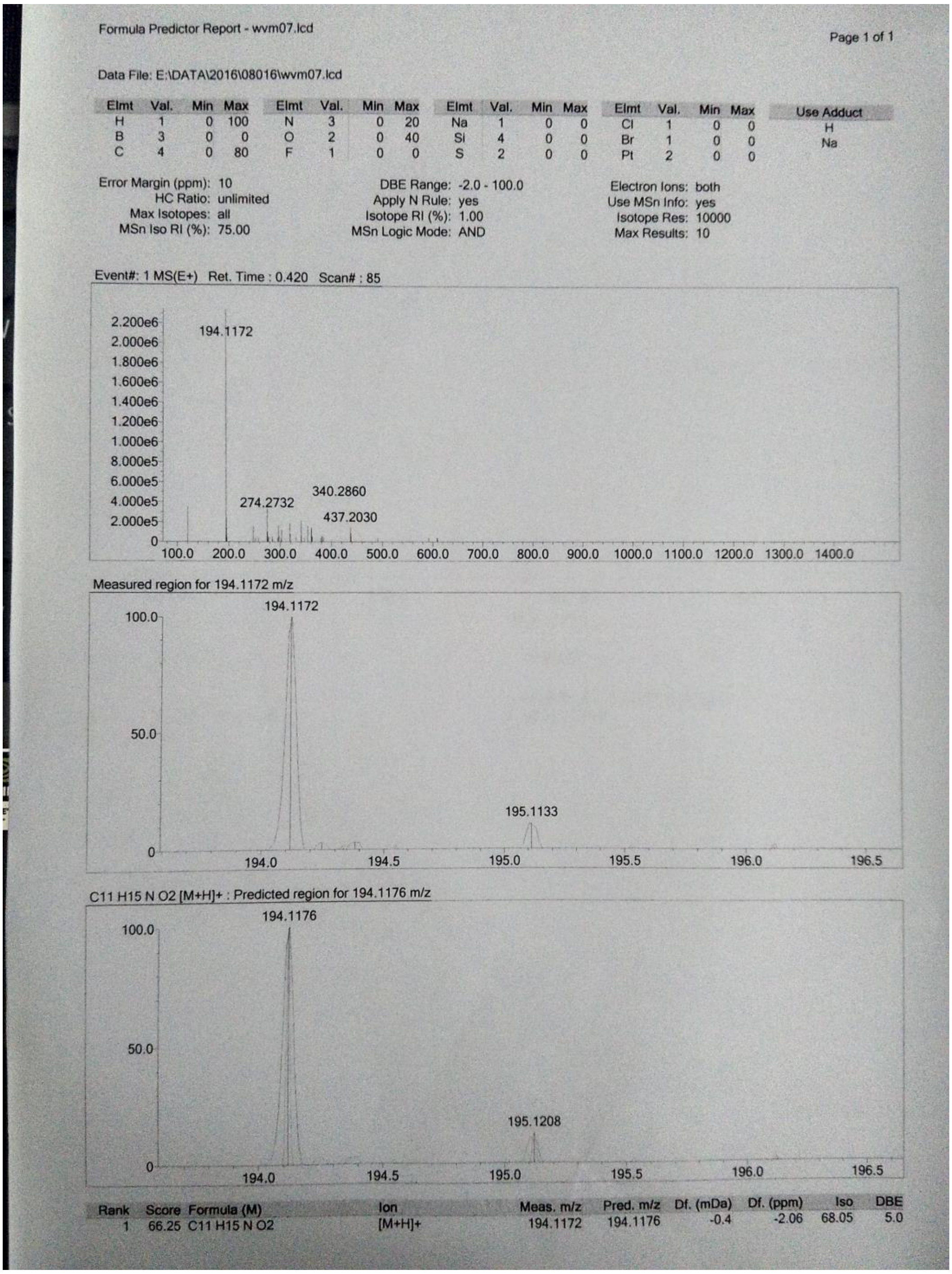

Formula Predictor Report - wvm07.lcd

Page 1 of 1

Data File: E:\DATA\2016\08016\wvm07.lcd

Elmt	Val.	Min	Max	Elmt	Val.	Min	Max	Elmt	Val.	Min	Max	Elmt	Val.	Min	Max	Use Adduct
H	1	0	100	N	3	0	20	Na	1	0	0	Cl	1	0	0	H
B	3	0	0	O	2	0	40	Si	4	0	0	Br	1	0	0	Na
C	4	0	80	F	1	0	0	S	2	0	0	Pt	2	0	0	

Error Margin (ppm): 10
HC Ratio: unlimited
Max Isotopes: all
MSn Iso RI (%): 75.00

DBE Range: -2.0 - 100.0
Apply N Rule: yes
Isotope RI (%): 1.00
MSn Logic Mode: AND

Electron Ions: both
Use MSn Info: yes
Isotope Res: 10000
Max Results: 10

Event#: 1 MS(E+) Ret. Time : 0.420 Scan# : 85

Measured region for 194.1172 m/z

C11 H15 N O2 [M+H]+ : Predicted region for 194.1176 m/z

Rank	Score	Formula (M)	Ion	Meas. m/z	Pred. m/z	Df. (mDa)	Df. (ppm)	Iso	DBE
1	66.25	C11 H15 N O2	[M+H]+	194.1172	194.1176	-0.4	-2.06	68.05	5.0

Figure 23S HRESIMS spectrum of compound **3**

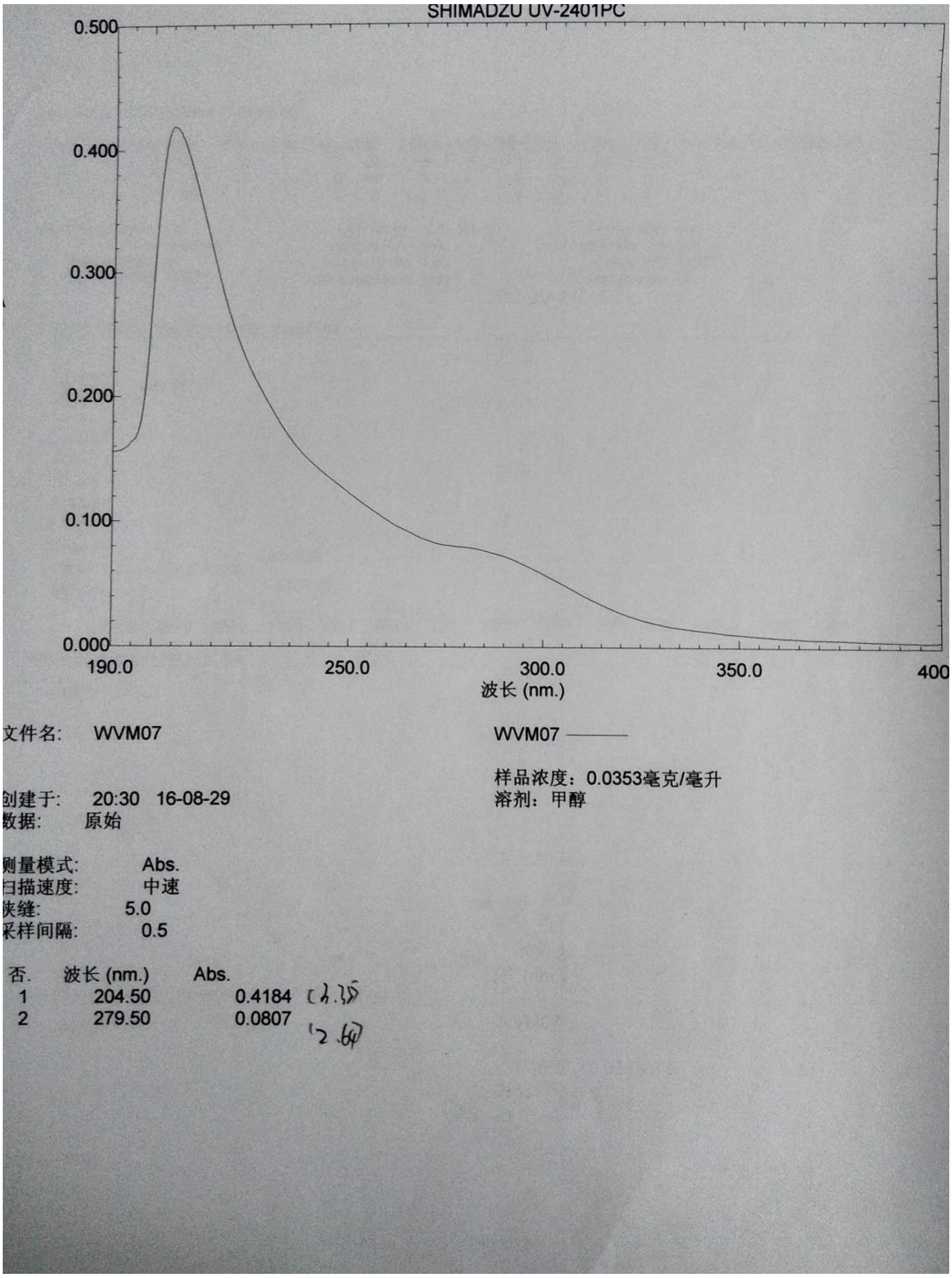

Figure 24S UV spectrum of compound **3**
